# A Case of Quadricuspid Aortic Valve Regurgitation: The Cardiac Clover

**DOI:** 10.1155/2021/5596297

**Published:** 2021-07-01

**Authors:** John S. Dayco, Shaun Cardozo

**Affiliations:** ^1^PGY-I, Internal Medicine, Department of Internal Medicine, Detroit Medical Center, USA; ^2^Division of Cardiology, Physician, Division of Cardiology, Detroit Medical Center, Wayne State University, Wayne State University Physician Group, USA

## Abstract

A quadricuspid aortic valve is a very rare congenital heart condition that can present as aortic regurgitation in the 5^th^ and 6^th^ decade of life. The following case report will describe a patient who presented with symptoms of severe aortic regurgitation and was found to have a quadricuspid aortic valve on echocardiography. The case will describe the clinical manifestations in which the patient presented and the subsequent diagnosis of the quadricuspid aortic valve. The rationale for the surgical approach will also be discussed, along with the patient's clinical response.

## 1. Introduction

A quadricuspid aortic valve (QAV) is a very rare congenital heart condition with an incidence of only 0.008-0.033% in an autopsy series of patients and 0.013-0.043% in patients undergoing transthoracic echocardiography (TTE) [1]. The pathogenesis is usually due to an abnormal fusion of the aorticopulmonary septum during embryogenesis, which leads to an aortic valve with an extra 4th cusp [2]. The complications manifested by QAVs are mainly aortic regurgitation, and a QAV is often incidentally discovered during an echocardiogram [3]. Aortic stenosis can also be observed along with regurgitation; however, aortic stenosis by itself is a rare presentation of a QAV. These complications usually arise during the 5^th^ to 6^th^ decade of life, as is observed in the following patient. This case report will highlight the nonspecific manifestation of a QAV, the classification with the Hurwitz and Roberts model, and the rationale for surgical intervention.

## 2. Case

**Figure 1:**
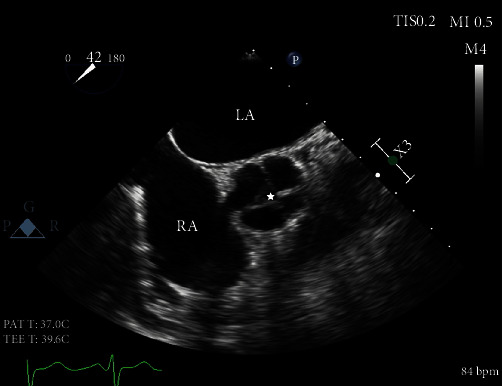
Mid epigastric short axis view. The closed aortic valve is demonstrating the characteristic X pattern as seen in QAVs in a TEE. The incomplete closure is marked with a star, leading to significant aortic regurgitation.

**Figure 2:**
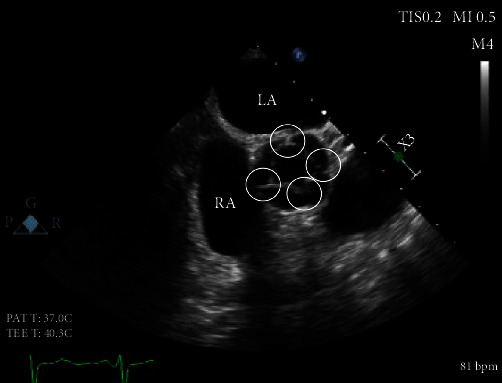
Mid Epigastric Short Axis View. During systole, 4 areas of commissural cusps fusion are noted (circled), suggesting aortic stenosis.

**Figure 3:**
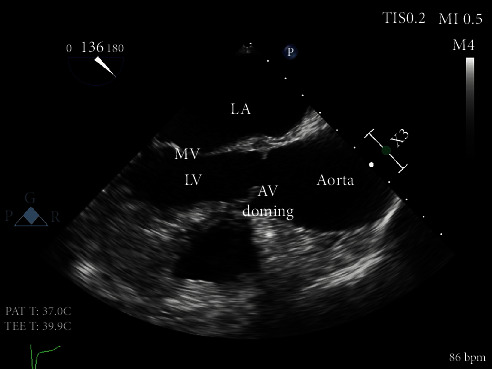
Mid epigastric long axis view. In Figure 3, aortic valve doming is seen during midsystole, suggesting asymmetry and restriction. In Figure 4, taken with doppler ultrasonography during middiastole, a “mosaic pattern” (star) is evident in the left ventricular area, suggesting aortic regurgitant flow.

**Figure 4:**
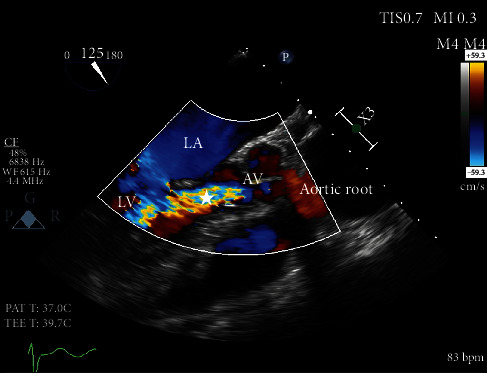
Mid epigastric long axis view. In Figure 3, aortic valve doming is seen during midsystole, suggesting asymmetry and restriction. In Figure 4, taken with doppler ultrasonography during middiastole, a “mosaic pattern” (star) is evident in the left ventricular area, suggesting aortic regurgitant flow.

**Figure 5:**
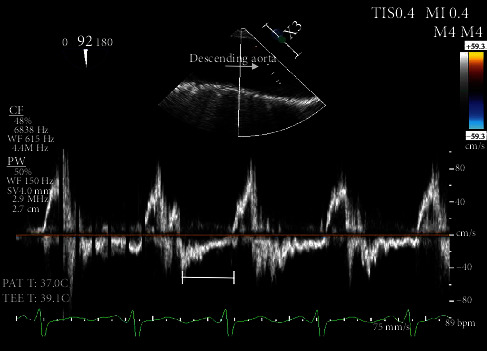
Descending aorta long axis view. In this image, a holosystolic aortic regurgitation is shown by the waveform labeled “I.”

Our patient is a 62-year-old female with a past medical history of heart failure with preserved ejection fraction (55-60% visually estimated ejection fraction) New York Heart Associate (NYHA) Class III, hypertension, and hyperlipidemia who was seen in clinic for worsening dyspnea on exertion. Her vital signs were stable, and on exam, she had a grade III/VI diastolic murmur along the left upper sternal border that increased with expiration. The EKG was normal sinus rhythm. ATTE was performed, which showed moderate to severe aortic regurgitation. In order to delineate the pathology and severity of the regurgitation, a transesophageal echocardiogram (TEE) was performed and showed an aortic valve morphology suspicious for a quadricuspid aortic valve (Figure 1). The valve demonstrated doming and four areas of commissural fusion were noted (Figure 2). The aortic root was mildly enlarged at 4.51 cm (Figure 3). There was holodiastolic flow reversal noted at the proximal descending aorta which helped to support severe regurgitation (Figures 4 and 5). The presence of a systolic aortic cusp doming also suggested a superimposed aortic stenosis (Figure 3). A coronary angiogram showed no significant coronary artery stenosis, and subsequently, the patient then underwent an aortic valve replacement (AVR) with a 21 mm Edwards Magna Ease bovine pericardial bioprosthetic valve. During the surgery, the presence of the quadricuspid aortic valve was confirmed on gross pathology. Immediately, postoperatively, the patient developed ventricular tachycardia and atrial fibrillation. She was then transferred to the surgical intensive care unit to receive an amiodarone drip for two days, which has resolved her atrial fibrillation and ventricular tachycardia. A repeat EKG showed normal sinus rhythm. She was then discharged with oral metoprolol. The patient underwent an uncomplicated recovery process, and her dyspnea on exertion has improved tremendously after a course of cardiac rehabilitation. A repeat TEE revealed a left ventricular end diastolic diameter of 4.7 cm, which is an improvement from 4.96 cm before the AVR, indicating a reduction in aortic regurgitation.

## 3. Discussion

This patient initially presented for dyspnea on exertion, along with a loud diastolic murmur in the left upper sternal border. While this kind of presentation is concerning for aortic regurgitation, the suspicion for a QAV did not arise until the patient received a TEE, which showed the characteristic “X” pattern of the aortic valve, as opposed to the normal “Y” (aka *Mercedes-Benz* pattern) of a normal tricuspid aortic valve (Figure 1). Aortic regurgitation is the most common complication of a QAV, and the finding of a holodiastolic flow reversal in the descending aorta supported this (Figure 5). Aortic stenosis could also be observed, such as when commissural fusion occurs (Figure 2). In Figure 4, the doppler image showed the characteristic “mosaic pattern,” as seen during the reversal of flow through the insufficient aortic valve in diastole. For the purpose of visualization and ease of access, cardiac echocardiography remains to be the imaging standard for a QAV. With the recent advent of more advanced imaging modalities, studies such as cardiac MRIs are proving to be particularly useful in the diagnosis of QAV. The gold standard in the classification of QAVs remains to be the Hurwitz-Robinson classification [2], which classifies QAVs into 7 different subtypes, depending on the cusp morphology (Table 1).

Types A, B, and C comprise the vast majority of cases at >80% [2]. Our patient, as seen in the images obtained in the TEE (Figure 1), has 3 identical cusps and 1 smaller cusp, classifying it as Type B.

The decision to perform a surgical aortic valve replacement on the patient was made on the basis of worsening dyspnea on exertion, with the QAV producing aortic regurgitation. By this time, the patient had progressed to New York Heart Associate Class III, exhibiting symptoms with mild exertion, such as going to the bathroom. Based on the ACC/AHA guidelines, AVR is indicated for symptomatic patients with severe AR regardless of LV systolic function. For such patients, immediate surgical intervention is indicated to greatly improve the patient's quality of life, as well as to prevent long-term sequela, such as left ventricular cavity dilation from the volume overload due to constant aortic regurgitation. In addition, a serious complication may also arise in which one of the supernumerary cusps may develop bacterial seeding, leading to infective endocarditis [4]. This usually occurs at the extra cusp, such as the one smaller cusp in Type B cases like our patient. Some authors recommend antibiotic prophylaxis unconditionally for any dental procedures [5]. However, per current ACC/AHA guidelines, antibiotics prophylaxis is only indicated for symptomatic patients with active infection [6]. The treatment of choice is surgical aortic valve replacement (SAVR) [7]. The surgical approach includes tricuspidization, bicuspidization, or a complete aortic valve replacement. These choices depend on factors such as the quality of the native aortic valve, the presence of calcification and stenosis, and surgeon capabilities. Our patient received a 21 mm Edwards Magna Ease bovine pericardial bioprosthetic valve, with a noncomplicated post-op recovery period. The SAVR procedure proved to be very effective for the patient, as a repeat TTE showed a significant improvement in the aortic regurgitation. More importantly, the shortness of breath and dyspnea on exertion has tremendously improved. The patient will require constant monitoring, such as a yearly TTE, due to the lower durability of bioprosthetic aortic valves as compared to a mechanical valve or native valve tricuspidization.

## Figures and Tables

**Table 1:** Hurtwitz-Robinson classification of quadricuspid aortic valve.

Type	Description	Morphology
Type A	(4) Identical cusps	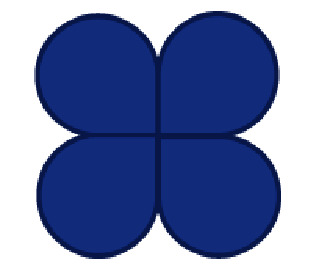
Type B	(3) Identical and (1) smaller cusp	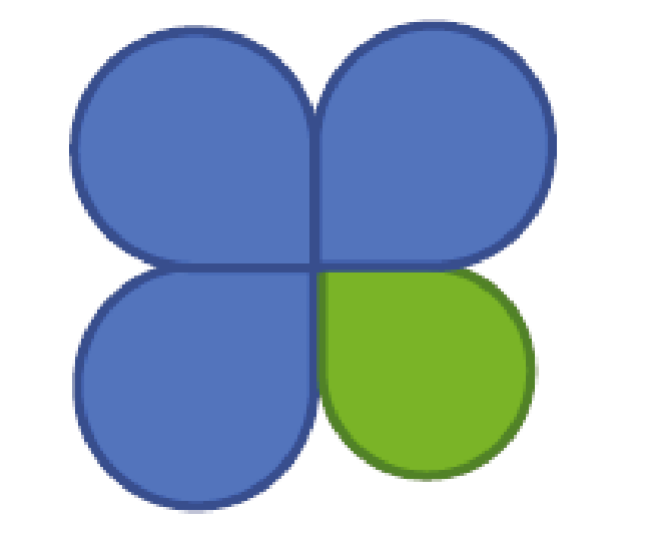
Type C	(2) Identical and (2) smaller cusps	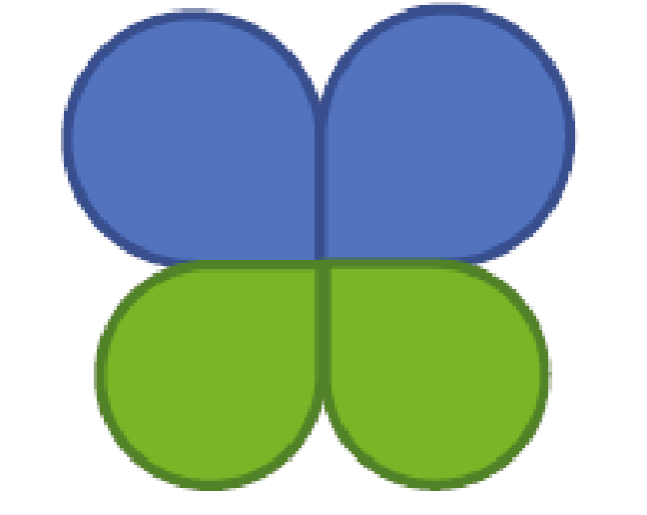
Type D	(1) Large, (2) intermediate, and (1) small cusp	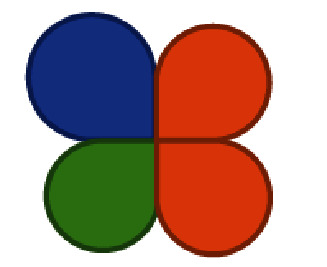
Type E	(3) Identical and (1) larger cusp	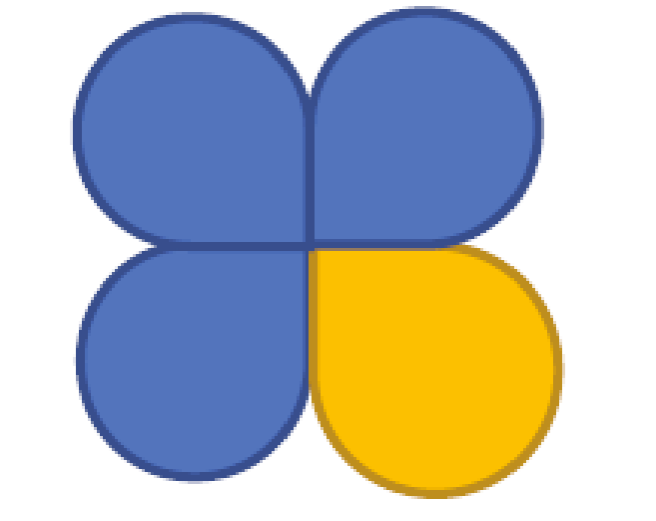
Type F	(2) Larger identical and (2) smaller nonidentical cusps	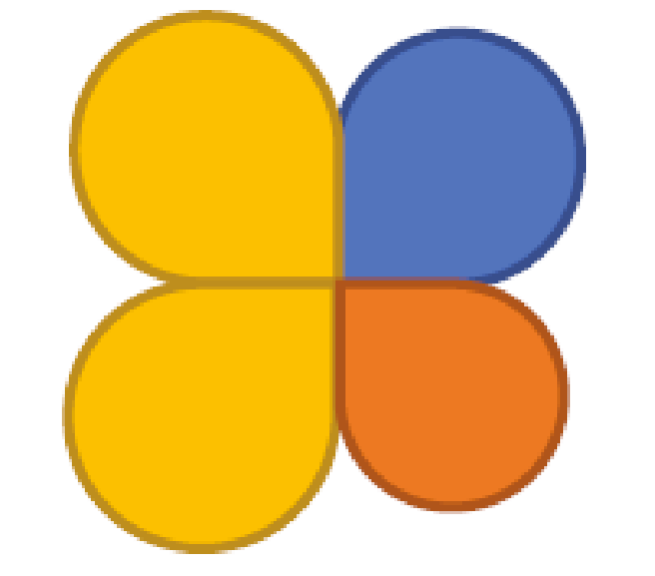
Type G	(4) Nonidentical cusps	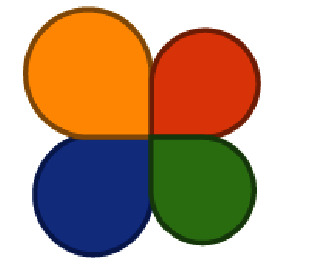

## Data Availability

The datasets used and/or analysed during the current study are available from the corresponding author upon request.
